# Periprosthetic Fractures around Total Hip Replacement—Is There a Rush to Fix?

**DOI:** 10.3390/jcm12103512

**Published:** 2023-05-17

**Authors:** Timothy Boddice, Peter Harrison, Christopher Anthony, Aaron B. Y. Ng

**Affiliations:** Pinderfields General Hospital, Aberford Road, Wakefield WF1 4DG, UK; peter.harrison8@nhs.net (P.H.); c.anthony2@nhs.net (C.A.); aaron.ng2@nhs.net (A.B.Y.N.)

**Keywords:** periprosthetic, hip, fracture, revision, early surgery

## Abstract

Background: Periprosthetic fractures now account for 14.1% of all hip revisions. Surgery is often highly specialised and can involve the revision of implants, fixation of fractures, or a combination of both. Delays to surgery are frequent as specialist equipment and surgeons are often required. UK guidelines are currently moving in favour of early surgery in a similar way to the neck of femur fractures, despite a lack of evidential consensus. Methods: A retrospective review of all patients who underwent surgery for periprosthetic fractures around a total hip replacement (THR) at a single unit between 2012 and 2019 was performed. Risk factors for complications, length of stay (LOS), and time to surgery data were collected and analysed using regression analysis. Results: A total of 88 patients met the inclusion criteria: 63 (72%) were treated with open reduction internal fixation (ORIF) and 25 (28%) underwent revision THR. Baseline characteristics were similar in both the ORIF and revision groups. Revision surgery was more likely to be delayed than ORIF owing to the need for specialist equipment and personnel (median 143 h vs. 120 h, *p* = 0.04). Median LOS was 17 days if operated within 72 h and 27 days if delayed beyond this (*p* < 0.0001), but there was no increase in 90-day mortality (*p* = 0.66), HDU admission (*p* = 0.33), or perioperative complications (*p* = 0.27) with delay beyond 72 h. Conclusion: Periprosthetic fractures are complex and require a highly specialised approach. Delaying surgery does not result in increased mortality or complications but does increase length of stay. Further multicentre research into this area is required.

## 1. Introduction

The combination of an aging population and good outcomes in arthroplasty surgery has led to an increase in the number of patients undergoing joint replacement surgery over the last decade. In 2019, the number of patients in the United Kingdom (excluding Scotland) undergoing primary total hip arthroplasty (THA) was 95,677—an increase of almost 40% over the last 10 years [1]. With the rise in frequency of THA and an increase in longevity, the numbers of periprosthetic fractures have also increased [2], with the UK National Joint Registry (NJR) reporting that periprosthetic fractures (PPFs) have been the indication for 14.1% of all hip revisions in the previous five years [1]. More recent work suggests that this is a significant underestimate, as NJR data do not include those treated with open reduction and internal fixation (ORIF) without implant exchange, which are estimated to be approximately 50% of cases [3].

The Best Practice Tariff (BPT) was introduced in England and Wales from 2010 and led to financial incentives being awarded if specific criteria were met in the management of proximal femoral fractures [4]. Since the introduction of the BPT, the 30-day mortality rate of proximal femoral fractures has fallen from 8.3% in 2009 to 6.1% in 2018 [5,6]. One criterion within the BPT is the timing of surgery to be within 36 h of presentation due to improved outcomes for such patients [4]. From January 2020, the BPT allows patients with PPFs to be recorded onto the database in the same way as proximal femoral fractures “in the expectation that in future years BPT will also be extended to these cases” [6]. Considering the likely inclusion of PPFs into the BPT in the near future, it is important to understand the effect of timing of operative intervention on patient outcomes. 

Periprosthetic fractures often occur in patients with multiple comorbidities who are high-risk surgical candidates [7,8,9]. This can result in a delay between presentation and definitive surgery to allow for medical optimisation. In addition, surgery is often complex, requiring both revision arthroplasty and trauma techniques to treat loose prostheses and bone loss as well as the fracture itself. Such surgery often falls outside the scope of a general orthopaedic trauma surgeon, and it is critical to ensure that the correct surgeon with the appropriate training and equipment is available [8,9,10,11]. Whilst the negative effects of a delay in surgery for the treatment of native femoral fractures have been well established, there is no such consensus with femoral periprosthetic fractures [7,8,9].

Despite this, there are definite similarities between proximal femoral fractures in the native hip and periprosthetic fractures. This is logical—both fractures occur in elderly, often unwell, patients and result in major surgery followed by reduced levels of mobility. Bhattacharyya et al. [12] found a similar 12-month mortality in both native hip fractures and periprosthetic fractures, while Haughom et al. [13] demonstrated a similar 30-day mortality and increased rates of complications in periprosthetic fractures.

There are conflicting results, however, when exploring the relationship between time to surgery and post-operative complications in patients with periprosthetic fractures around the hip, and a growing body of evidence that there is no increase in mortality with delayed surgery, unlike in native proximal femoral fractures. 

A prolonged time between injury and operative fixation is not necessarily associated with increased rates of mortality [7,8,9,10,11,14]. However, there is conflicting evidence as to whether there are increased rates of complications in patients who wait for fixation [7,8,9,14], and it remains uncertain as to why the relationship between time to surgery and mortality is not established as it is in native hip fractures. 

Two large registry studies in the USA found no increase in mortality with a delay of greater than 24 h or 48 h [8,11], although they differed on whether there was an increased complication rate. Other studies reported similar findings, with no difference in mortality but increased complication rates with delay [2,14], while others reported no difference in either mortality or complications [9,10]. A systematic review in 2020 [7] found a small increase in 30-day mortality but no difference at 12 months, and also found that many of the studies were of insufficient quality to draw firm conclusions.

Our study aims to add to the available evidence base. We review whether the timing of surgery following a periprosthetic hip fracture influences post-operative outcomes in our unit, by examining length of inpatient stay, morbidity, and mortality. Our null hypothesis is that periprosthetic fractures are similar to native hip fractures and will demonstrate similar patterns of increasing morbidity and mortality with surgical delay.

## 2. Methods

A retrospective observational study of consecutive patients was conducted within a large district hospital trust in the United Kingdom. A search was undertaken for all patients who had undergone surgical fixation or revision for a femoral periprosthetic fracture between the years 2012 and 2019 using the Bluespier^TM^ theatre management system (Bluespier International, Droitwich, UK). Codes searched for were as following: Closed Periprosthetic Femoral Fracture (S72.90), Closed Periprosthetic Fracture of the Proximal Femur (S72.20), Open Periprosthetic Femoral Fracture (S72.91), Open Periprosthetic Fracture of the Proximal Femur (S72.21), Periprosthetic Fracture of the Femoral Stem of Hemiarthroplasty (S72.20), ORIF Distal Periprosthetic Femoral Fracture, ORIF Proximal Periprosthetic Femoral Fracture, Revision THR for Periprosthetic Fracture, and THR for Conversion of Hemiarthroplasty Periprosthetic Fracture. 

**Figure 1:**
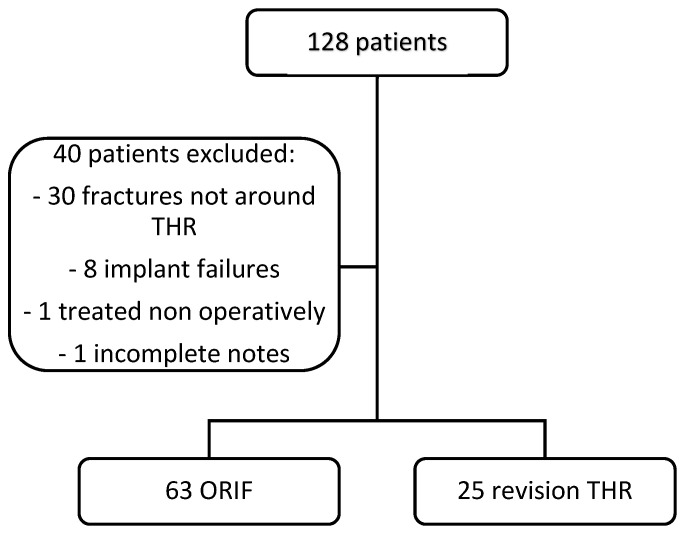
Patient selection and exclusion criteria. The flow of patients included in the study is shown. (THR = Total Hip Replacement, ORIF = Open Reduction Internal Fixation).

All patients who had undergone surgical fixation or revision surgery for a fracture around a proximal femoral prosthesis were included in the sample. Inclusion criteria included the presence of a total hip replacement prior to fracture, a procedure in theatre involving fixation, or revision surgery within the specified date range. Prosthetic neck and stem fractures were excluded. Once the sample was identified, case note review was undertaken using X-rays, electronic notes, and clinic letters to confirm that patients met the inclusion criteria and the necessary data was gathered. Exclusion criteria are shown in Figure 1.

The time of the first radiological evidence of a periprosthetic fracture was used to determine the time of diagnosis, whilst the time of anaesthetic induction was used to determine the time of surgery.

Patient characteristics such as age, gender, American Society of Anaesthesiologists (ASA) grade, smoking status, and diabetes mellitus were collected from a review of case notes. A review of pre-operative comorbidities was used to calculate individual Charlson comorbidity index scores to stratify the mortality risk of the cohort. Post-operative outcome measures included urinary and respiratory tract infections, intensive care unit stay, myocardial infarction, wound infection, deep vein thrombosis, pulmonary embolism, and repeat operation. The presence of mechanical compilations, joint infection, and mortality were also measured at 90 days.

Patients were placed into two cohorts based on the time from diagnosis to surgery, either <72 h or >72 h. A 72 h time period was chosen in order to cover a weekend period when specialist expertise or equipment may not be available.

Statistics were compiled using Graphpad PRISM version 9, with statistical significance defined as *p* < 0.05. Outcomes between the two cohorts were assessed using bivariate and multivariate analyses. Categorical data were assessed for statistical significance using Fisher’s Exact Test. Continuous data were assessed for normality using a Shapiro–Wilk test. If found not to be normally distributed, an attempt was made to normalise the data using a natural log transformation. Normal data were analysed using an unpaired two-tailed T-test. Nonparametric data were subjected to a Mann–Whitney U test. The authors declare that there are no conflicts of interest and that they received no funding. This study was performed under local governance approval.

## 3. Results

A total of 128 patients were identified from the initial search. Of these, 40 were excluded as above in Figure 1. Of the remaining 88 patients, 63 (72%) underwent ORIF and 25 (28%) underwent revision total hip replacement. The mean age was 77.01 years (95% CI 74.62–79.41), with there being 22 males and 66 females. The mean Charlson comorbidity index (CCI) across the cohort was 4.489 (95% CI 4.039–4.938). There was no significant difference in baseline characteristics between the ORIF and revision groups (Table 1).

ORIF had a significantly shorter time to surgery compared to revision, 120.45 h vs. 143.78 h (*p* = 0.04). There was no difference in length of stay or post-op length of stay between the patients receiving ORIF vs. revision surgery (*p* = 0.38 and *p* = 0.5353, respectively), as shown in Table 2.

A total of 21 patients (24%) received surgery within 72 h of admission. The median length of stay was 10 days shorter in those who received surgery within 72 h, and this was statistically significant (*p* < 0.0001). In addition, the median length of post-operative stay was 7 days shorter in this group (*p* = 0.014). No statistically significant difference was found in terms of mortality (*p* = 0.67), need for high-dependency post-operative care (*p* = 0.33), or systemic complications (*p* = 0.27) between the groups, as shown in Table 3.

Linear regression showed a correlation between time to surgery and length of stay (R2 = 0.07597, *p* = 0.0093) but time to surgery and post-operative length of stay did not reach significance (R2 = 0.00265, *p* = 0.63), suggesting that much of the increased length of stay was waiting for surgery rather than a prolonged recovery. Logistic regression did not show any significant association between time to surgery and mortality (R2 = 0.006443, *p* = 0.432). Multiple regression did not show any significant relationship between age (*p* = 0.489), operation performed (*p* = 0.204), ASA (*p* = 0.2245), or CCI (*p* = 0.472) with length of stay. Being male was associated with a longer wait for surgery (*p* = 0.029).

There was no significant increase in mortality resulting from a wait of more than 72h for surgery, with a relative risk of 0.7836 (95% CI 0.1639–3.7474). The recoveries of 12 patients were complicated by post-operative myocardial infarctions, hospital-acquired pneumonias, or urinary tract infections. Waiting more than 72 h for surgery did not significantly increase these complications, with a relative risk of 3.4478 (95% CI 0.4724–25.1618). From the ORIF group, one patient (67 h) suffered a pulmonary embolus, one patient (37 h) suffered a deep infection treated with washout in theatre, and one patient (187 h) suffered a post-operative dislocation treated with subsequent revision arthroplasty.

If a shorter time of 48 h was used to split the groups, this would confirm the reduced length of stay with earlier surgery (median 15 vs. 26 days, *p* = 0.0005). It does not result in any significant difference in morbidity or mortality despite there being no deaths of patients operated on within 48 h, which is likely due to the low numbers in this group (n = 11).

## 4. Discussion

This study echoes similar studies, demonstrating no increased morbidity or mortality with delayed surgery for periprosthetic fractures beyond 72 h. Although the length of overall stay is increased with delayed surgery beyond 72 h, a delay to allow medical optimisation and preparation for a specialised definitive operation is reasonable and not associated with increased complications.

Whilst there has been a clear correlation established in the literature between time to surgery in native hip fractures with post-operative outcomes [4], this relationship has not been consistently proven in periprosthetic hip fractures. Considering the growing consensus in the literature, it seems that unlike native hip fractures, a prolonged time between injury and operative fixation is not necessarily associated with increased rates of mortality [7,8,9,10,11,14]. However, there is conflicting evidence as to whether there are increased rates of complications in patients who wait for fixation [7,8,9,14], and it remains uncertain as to why the relationship between time to surgery and mortality is not established as it is in native hip fractures. 

Recently, there have been two large studies examining the impact of timing of PPF surgery using national registry data in the USA. Boddapati et al. [8] examined the records of 857 patients from the American College of Surgeons National Surgical Quality Improvement Program (ACS-NSQIP) who had undergone surgical fixation for a periprosthetic fracture and divided them into “expedited” (definitive surgery within 24 h of admission) or “non-expedited” (definitive surgery after 24 h), with outcome measures of readmission, morbidity, mortality, complications, and transfusion rates. The authors concluded that although there was a “strong trend in favour of expedited surgery”, there was not a significant difference in mortality [8], although there were increased rates of morbidity and complications at 30 days within the delayed surgery group, including respiratory complications, urinary tract infections, and an increased length of post-operative hospital stay [8]. 

In a 681-patient study, Bovonratwet et al. [11] examined the same registry with groups divided by whether surgery was performed within 2 days or later and determined that there was no relationship between delayed surgery and mortality, post-operative event, or post-operative complications. In keeping with Boddapati et al. [8], they found that surgery after the 2-day period had a higher risk of a prolonged length of post-operative inpatient stay. Both studies were based on retrospective registry data and the authors of both papers noted that the expedited group was, on average, younger and healthier and had a shorter average length of procedure under general anaesthesia, introducing a potential bias to the results.

Scott et al. [14] used a different registry (Medicare) to retrospectively look at 597 patients, and like Bovonratwet et al. [11], used 48 h as a cut-off point for early or delayed surgery. They concluded that there was an increased rate of PE or DVT, an increased risk of re-presentation to the emergency department, and an increased risk of infection in those who had surgery more than 48 h after presentation [14]. This study, however, did not look at the association between delayed surgery and mortality, and is again prone to the bias of retrospective analysis, although they reported no difference between the early and delayed surgery groups in age or comorbidity. 

Farrow et al. [7] performed a systematic review and meta-analysis of the literature in 2020, looking at time to surgery. They demonstrated a slight increase in 30-day mortality but no increase in 12-month mortality on meta-analysis, but with the caveat that the data were subject to confounding and of generally limited quality owing to the retrospective study design. They also reported increased morbidity and complication rates with surgical delay, although, again, low numbers and inconsistent methodology between studies caused them to recommend interpreting the results with caution and recommending a large-scale or registry- based research with a minimum data-set.

A Canadian study by Sellan et al. [10] included 180 patients at a single institution who had a periprosthetic fracture around THA and total knee arthroplasty (TKA). Similarly, to the above studies, patients were dichotomised at 48 h from presentation to surgery. Waiting longer than 48 h for surgery did not affect the rate of complications, length of post-operative stay, and mortality at both 30 days and 1 year—however, this included both hips and knees and did not stratify by whether ORIF or revision arthroplasty was performed [10]. 

An 82-patient study conducted by Johnson-Lynn et al. [9] in two NHS Foundation Trusts in the United Kingdom also concluded that there was no association between delayed surgery and both inpatient and 1-year mortality [9]. This study took a different approach and did not dichotomise patients into “early” and “delayed”, but instead used statistical correlation to examine the relationship between operative time and time to surgery with complications and mortality. A further single-centre study consisting of 60 patients by Griffiths et al. [2] found that a delay of more than 72 h to surgery was associated with an overall increase in the risk of complications but no significant difference in mortality.

With the number of published studies including this study, it seems unlikely that a significant increase in morbidity or mortality would have been overlooked, although as Farrow [7] suggests, this may be due to a lack of statistical power or limitations of methodology. Given the lack of a meaningful association between mortality and time to surgery demonstrated so far, a comparison must be made with those with native hip fractures to determine a potential explanation for these findings.

In both this study and others, the patient cohort is not dissimilar to that expected in patients with native hip fractures. Our patient group had an average age greater than 75 years, and the majority of patients were ASA grade 3 or higher with several comorbidities, undergoing major surgery following trauma, and significant injury. 

Hip fractures often occur following very low energy trauma in a patient with reduced bone density [15], and in periprosthetic fractures, the altered biomechanics attributed to a prosthesis may reduce this association. Evidence has shown different rates of periprosthetic fractures for different designs of femoral stem [16], and therefore it may be inferred that the presence of a prosthesis changes both the energy required to fracture as well as the morphology and location of the fracture. Patients undergoing fixation without revision for periprosthetic fracture (more than 70% of our cohort) are less likely to have femoral canal instrumentation or cementation, both of which increase mortality in the short term [17], and the absence of this physiological insult may account for some of the reduction in morbidity and mortality.

Early mobilisation is the standard of care in hip fracture patients and whilst it is also recommended in periprosthetic fractures [4,18], there is strong evidence that this is often disregarded in favour of protected or non-weight bearing activity, particularly following ORIF [19]. Whilst we have not formally examined this here, it does not appear to have made a significant difference assuming that the revision arthroplasty group mobilised full-weight bearing, as we observed no difference in mortality between the ORIF and revision groups.

As with hip fractures, there is often a desire to delay surgery for medical optimisation for theatre, and this may account for some of the delay in this group. Changes in NICE guidance for hip fractures and the resulting outcomes [4,6] show that for hip fractures, early surgery is beneficial in terms of morbidity, mortality, and length of stay. However, periprosthetic fractures are more complex and require highly specialised management, which may not always be available in the first 24–48 h and may require additional medical optimisation given the longer and more complex procedures required. Delays to allow the deployment of specialist surgeons and equipment may be necessary, and may also be unavoidable without considerable reorganisation of services to accommodate this in the context of an increasing prevalence of periprosthetic fractures. It is our opinion that a delay to obtain the right expertise, equipment, and supporting team leads to the best outcome for the patient, and this study would support that view. 

This study’s main limitation is similar to many other studies, as the patient cohort was retrospectively gathered from one NHS Trust and represents a small but heterogeneous cohort with a significant risk of confounding. In particular, the number undergoing revision surgery within 72 h is very small (*n* = 4) and we would caution against changing practice based on this, although this fits with the rest of our findings that delay makes no difference to mortality or complications. However, we believe that this study accurately reflects the diversity of patients presenting to the department over a long period of time with its broad inclusion criteria, and therefore lends itself to application to a wide and diverse population. Although a single-centre study, this is a large sample size for a single trust which encompasses three separate hospital sites across a population of more than 500,000. Although there is a risk of type 2 error, as with other studies with small sample sizes and methodologies, the number of other studies with similar findings suggests that this is not the case. Large-scale multicentre research would be required to provide definitive evidence and minimise the risk of confounding.

## 5. Conclusions

There is no evidence of increased morbidity, complications, or mortality with delayed surgery for periprosthetic fractures beyond 72 h in our cohort. Although length of overall stay is increased with delayed surgery beyond 72 h, a delay to allow medical optimisation and preparation for a specialised definitive operation is reasonable and not associated with increased complications.

## Figures and Tables

**Table 1:** Baseline characteristics of included patients, stratified by treatment.

	ORIF (*n* = 63)	Revision (*n* = 25)	*p*-Value
**Mean Age (years)**	76.59 (95% CI 73.52–79.65)	77.2 (95% CI 73.73–80.67)	*p* = 0.8183 ^†^
**Sex M:F**	1:3.5	1:2.125	*p* = 0.4148 *
**ASA 1 (%)**	3.17	0	*p* > 0.9999 *
**ASA 2 (%)**	33.34	44	*p* = 0.4616 *
**ASA 3 (%)**	50.79	40	*p* = 0.4785 *
**ASA 4 (%)**	12.70	16	*p* = 0.7349 *
**Charlson comorbidity index**	4.57 (95% CI 4.02–5.12)	4.6 (95% CI 3.74–5.46)	*p* = 0.9554 ^†^

* = Fisher’s Exact Test. ^†^ = Mann–Whitney U Test.

**Table 2:** Time to surgery and outcomes by treatment type.

	ORIF	Revision	*p*-Value
**Mortality**	7.94%	7.69%	*p* > 0.9999 *
**Median time to surgery (H)**	120.45 (95% CI 89.37–138.33)	143.78 (95%CI 111.93–184.47)	*p* = 0.0443 ^‡^
**Surgery within 72 h**	25%	16%	*p* = 0.4161 *
**Median length of stay (D)**	22 (95% CI 18–26)	27 (95% CI 18–35)	*p* = 0.3879 ^†^
**Median length of post-op stay (D)**	14 (95% CI 14–21)	14 (95% CI 14–26)	*p* = 0.5353 ^‡^

* Fisher’s Exact Test. ^†^ Mann–Whitney U Test. ^‡^ *t*-Test following ln conversion.

**Table 3:** Table comparing outcomes in patients who received their surgery before or after 72 h from admission.

	<72 h (*n* = 21)	>72 h (*n* = 67)	*p*-Value
**Mortality**	9.52% (*n* = 2)	7.46% (*n* = 5)	*p* = 0.6699 *
**Median length of stay (D)**	17 (95% CI 9–20)	27 (95% CI 25–33)	*p* < 0.0001 ^‡^
**Median length of post-op stay**	14 (95% CI 7–17)	21 (95% CI 17–25)	*p* = 0.014 ^†^
**Post-op HDU/ITU**	0% (*n* = 0)	7.46% (*n* = 5)	*p* = 0.3323 *
**Post-op MI/CAP/UTI**	4.76% (*n* = 1)	16.42% (*n* = 11)	*p* = 0.2744 *

* Fisher’s exact test. ^†^ Mann–Whitney U Test. ^‡^ *t*-test following ln conversion.

## Data Availability

Data is not publicly available due to privacy and data protection legislation. This paper represents an analysis of confidential patient records. Limited and anonymised data may be shared on request to the corresponding author.
